# Contrasting volcano spacing along SW Japan arc caused by difference in age of subducting lithosphere

**DOI:** 10.1038/s41598-020-72173-6

**Published:** 2020-09-14

**Authors:** Yoshiyuki Tatsumi, Nobuaki Suenaga, Shoichi Yoshioka, Katsuya Kaneko, Takumi Matsumoto

**Affiliations:** 1 Kobe Ocean-Bottom Exploration Center, Kobe University, Kobe, 658-0022 Japan; 2 Department of Planetology, Kobe University, Kobe, 657-8501 Japan; 3 Research Center for Urban Safety and Security, Kobe University, Kobe, 657-8501 Japan; 4 National Research Institute for Earth Science and Disaster Resilience, Tsukuba, 305-0006 Japan

**Keywords:** Tectonics, Volcanology, Geophysics

## Abstract

The SW Japan arc built by subduction of the Philippine Sea (PHS) plate exhibits uneven distribution of volcanoes: thirteen Quaternary composite volcanoes form in the western half of this arc, Kyushu Island, while only two in the eastern half, Chugoku district. Reconstruction of the PHS plate back to 14 Ma, together with examinations based on thermal structure models constrained by high-density heat flow data and a petrological model for dehydration reactions suggest that fluids are discharged actively at depths of 90–100 km in the hydrous layer at the top of the old (> 50 Ma), hence, cold lithosphere sinking beneath Kyushu Island. In contrast, the young (15–25 Ma) oceanic crust downgoing beneath Chugoku district releases fluids largely at shallower depths, i.e. beneath the non-volcanic forearc, to cause characteristic tectonic tremors and low-frequency earthquakes (LFEs) and be the source of specific brine springs. Much larger amounts of fluids supplied to the magma source region in the western SW Japan arc could build more densely-distributed volcanoes.

## Introduction

Subduction zone volcanoes tend to exhibit regular spacing along a volcanic arc, although the spacing of volcanoes within individual arcs is often variable from arc to arc^1, 2^. A broad positive correlation between the linear density of active volcanoes and the rate of plate convergence suggests that the faster subduction contributes to greater melt production in the mantle wedge^3–5^. Given that the fluids discharged from the subducted lithosphere drive magma generation, then a greater fluid flux is likely to enhance melt generation and may ultimately be linked to larger volcano numbers through increased formation rate of gravitational instabilities within the partially molten region in the mantle wedge^4, 6–8^.

**Figure 1:**
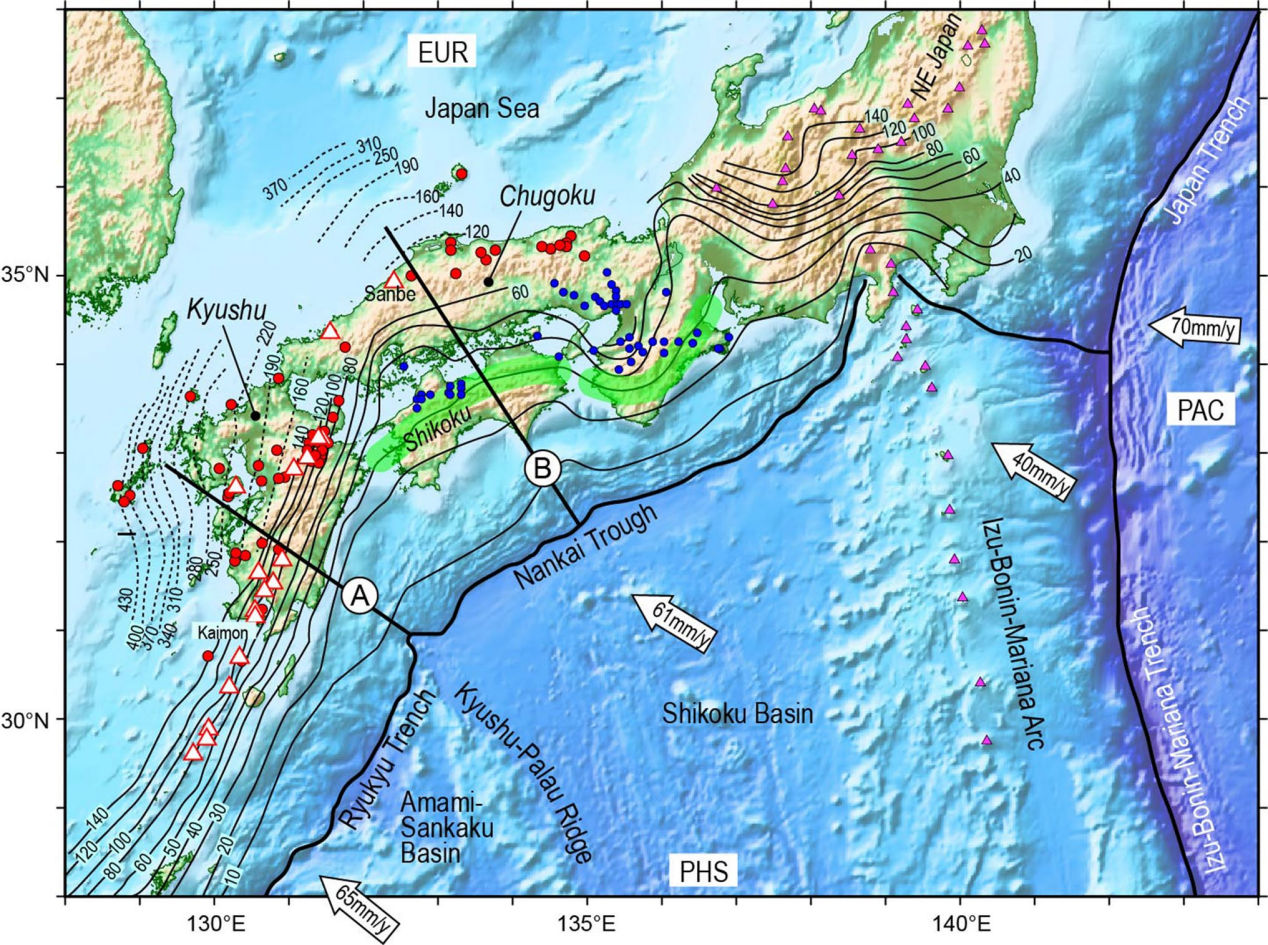
Tectonic setting of the western part of the Japanese Archipelago, which is an orogenic belt activated by subduction of both the Pacific (PAC) and the Philippine Sea (PHS) plates beneath the Eurasian (EUR) plate building active volcanoes on the NE Japan and Izu-Bonin-Mariana (purple triangles) and the SW Japan arcs (white triangles), respectively. Quaternary volcanoes in the SW Japan arc are also shown by red circles. The thin and solid continuous lines denote depth contours to the top of the subducted PHS slab estimated based on the seismicity in the PHS slab and local-earthquake tomography^14^ and the broken lines show those estimated based on the teleseismic tomography^14^. Blue circles and a green belt indicate distributions of high Li/Cl brine springs and a LFE zone^21^, respectively. The thermal structures were estimated along the profiles A and B.

In addition to the rate of subduction, the slab temperature should also impinge on volcano distribution in arcs. The contrasting volcano density observed in the Japanese Archipelago may be attributed to the difference in slab temperature: the downgoing Pacific plate beneath NE Japan where active volcanoes are densely distributed is much older (ca. 200 my) and cooler than the PHS plate beneath SW Japan^9^. The abundant arc volcanism in NE Japan reflects partial melting in the overlying mantle wedge, triggered by active infiltration of slab-derived fluids, while most of the water in the warm PHS plate is driven off at shallow depths and is not available to trigger effective magma production in the mantle wedge beneath SW Japan^9^. This pioneering work estimated thermal structure of the PHS plate beneath the eastern part of SW Japan, the Chugoku district, where active volcanoes are sparsely distributed (Fig. 1). The western part of SW Japan, the Kyushu region is, on the other hand, a volcanic arc exhibiting higher linear density of active volcanoes, in marked contrast to the Chugoku region of this arc, although both regions are underlain by the lithosphere of the PHS plate (Fig. 1). It should be here stressed, however, that the age of the PHS plate differs contrastingly, 25–15 vs. ~ 50 my for lithospheres of the western eastern part of the PHS plate, respectively^10–12^; the Kyushu-Palau Ridge (KPR) forms the boundary between these young and old lithospheres (Fig. 1). The difference in age hence temperature of the subducting slab could cause the contrasting volcano spacing along the SW Japan arc. Herein this hypothesis will be discussed based on the age of the subducted PHS plate inferred by reconstruction of plate motion, and calculation of temperature distribution along the sinking PHS plate beneath the Chugoku and Kyushu regions, and the behavior of water during subduction of the PHS plate.

### Tectonic setting of the SW Japan arc

The PHS plate is being subducted beneath the Eurasian (or Amurian) plate along the Nankai Trough and the Ryukyu Trench at a rate of 40–70 mm/year^13^, in the northwest direction to form the SW Japan arc (Fig. 1). Earthquakes in the PHS slab take place actively down to a depth of ∼ 150 km under Kyushu Island and ∼ 80 km beneath the Chugoku region^14^ (Fig. 1). The PHS slab has been further sinking aseismically down to a depth of ∼ 400 km^15, 16^ (Fig. 1). Tectonic tremors and LFEs, which may be caused by fluid activity associated with dehydration of the downgoing slab^17, 18^, have been identified beneath the non-volcanic forearc along the surface of the subducted PHS slab at depths of approximately 30–40 km with a belt-like along-arc distribution (Fig. 1). LFEs occur in high ${V}_{P}/{V}_{S}$ areas, indicating the existence of fluid, near the plate boundary between the down-dip end of the locked seismogenic zone of megathrust earthquakes and the up-dip end of the stable sliding region in western Shikoku^19^.

Active arc volcanoes that are composed of lavas and volcaniclastics having the calc-alkaline signatures form 100–200 km above the top of sinking PHS plate in SW Japan, though the volcano density changes markedly between the Kyushu and the Chugoku segments (Fig. 1).

In the non-volcanic forearc of the eastern half of this arc spring out characteristic deep-seated fluids referred to as the Arima-type brines (Fig. 1) possessing high Cl contents, high Li/Cl ratios, specific δ^18^O–δD isotopic ratios, and high ^3^He/^4^He ratios^20, 21^. Such geochemical characteristics of these brines may be attributed to dehydration of the downgoing PHS oceanic crust^21–24^.

The Japan Sea behind the SW and NE Japan arcs (Fig. 1) is a backarc basin created 30 to 15 Ma by rifting of the eastern margin of the Asian continent^25^. The opening of this backarc basin caused clockwise and counterclockwise rotations of the SW and NE Japan arc slivers, respectively, at **∼ **15 Ma^26^.

South of SW Japan is the Shikoku Basin (Fig. 1), which is also a backarc basin that formed behind the Izu-Bonin-Mariana arc by rifting 25 to 15 Ma^10^. Through this backarc rifting, the Izu-Bonin-Mariana arc sliver was separated from the paleo-Kyushu-Palau arc and migrated eastward, creating a new oceanic crust of the Shikoku Basin. It is thus inferred that the southward drift of the SW Japan arc, in association with both the Japan Sea opening and the clockwise rotation of the arc sliver, resulted in enforced subduction of the young (< 15 my) hence warm oceanic lithosphere of the Shikoku Basin beneath the eastern half of the SW Japan arc. The Amami-Sankaku Basin behind the KPR on the PHS plate (Fig. 1) was born > 48.7 Ma by backarc spreading within a Cretaceous-age island arc system^11, 12^ and is being subducted beneath the southern part of the SW Japan arc at a rate of > 65 mm/years (Fig. 1). It should be again noted that the age of the PHS plate changes greatly, 25–15 vs. ~ 50 my, across the KPR^10–12^.

## Results and discussions

### Volcano distribution in the SW Japan arc

Contrasting active volcano spacing is a characteristic in the SW Japan arc (Fig. 1). Identification of volcano spacing based solely on active Holocene volcanoes, however, may mislead the time scale of dynamic processes operating in the subarc mantle. The reasons for believing so are twofold. Firstly, although mafic melts can get transferred from source in the mantle wedge to surface rapidly, possibly within less than ~ 1 ky, magmatic differentiation from basalt to evolved andesite magmas that are the major volcanic products at convergent margins may need much longer time, i.e., > 100 ky^27^. Secondly, large volcanoes in the Japanese Archipelago (> 40 km^3^) have continued activity for 400–1,300 ky^28^, suggesting that the life span of arc composite volcanoes may be several hundreds of kilo years. In order to examine the linkage between plate subduction and volcano formation, therefore, distribution of Quaternary volcanoes in the SW Japan, rather than that of active Holocene volcanoes, should be examined, because these volcanoes may be built by current motion of the PHS plate that have been constant since 3 Ma as described later. Figure 1 clearly exhibits that Quaternary volcanoes are much more densely built in the eastern half of this arc, although only two active volcanoes exist there.

**Figure 2:**
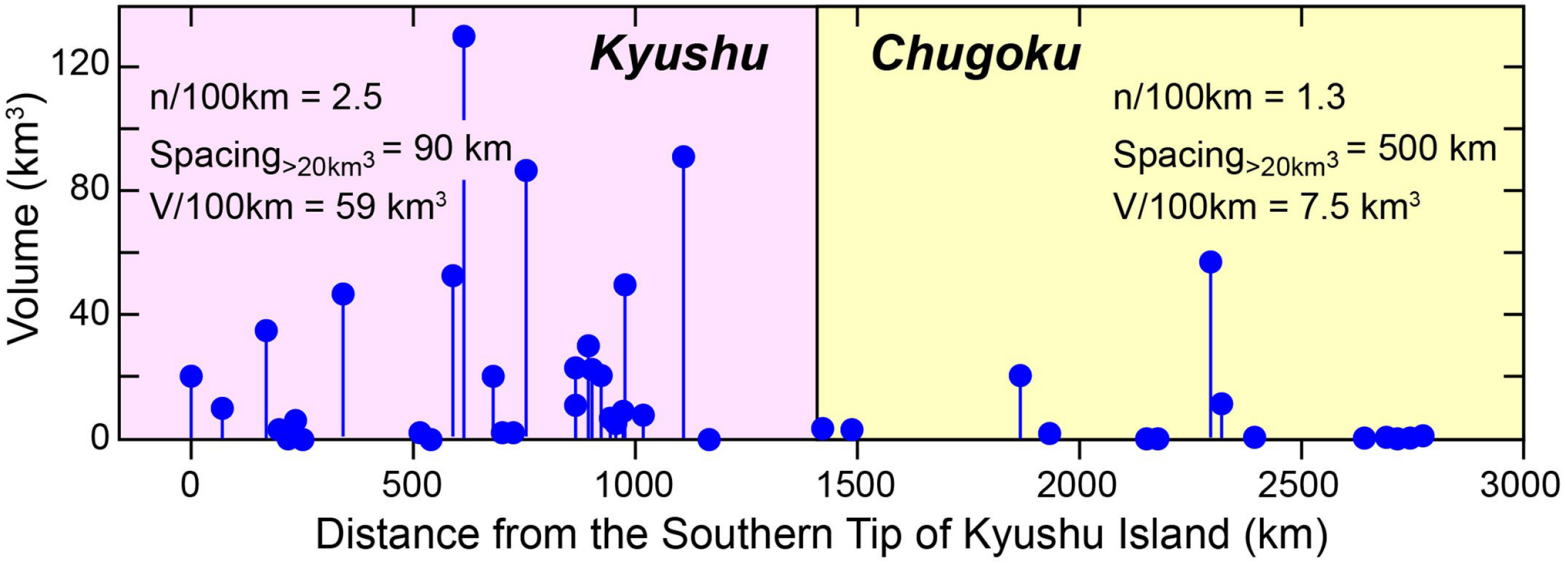
The volume and location of Quaternary volcanoes along the SW Japan arc from Kaimon volcano at the southern tip of Kyushu Island^29^. Contrasting volcano spacing and volume of volcanics are observed for Kyushu and Chugoku regions. In case that volcanoes form behind the volcanic front in the Kyushu region, the locations of such volcanoes are projected onto the volcanic front. In this diagram, volcanoes along the northern margin and on islands to the north and west of Kyushu Island are not included, because they are composed of alkaline basalts and may not be produced in association with subduction of the PHS plate. Some volcanoes in the SW Japan arc such as Aira and Aso volcanoes in Kyushu and Daisen in Chugoku erupted large amounts of felsic ignimbrites and/or tephra. These voluminous felsic magmas are not considered in this figure, since they may be produced by anatexis of the crust, not by differentiation of mantle-derived magmas^30^.

Figure 2 shows the along-arc distribution of Quaternary volcanoes and the volume of each volcano. It should be stressed in this diagram that most Quaternary volcanoes in the Chugoku region are small and form monogenetic volcanoes^31, 32^. As a result of this, total volume of volcanics distributed in the Kyushu region is ~ 7 times larger than in the Chugoku district. Furthermore, the number of large volcanoes (> 20 km^3^) is thirteen in Kyushu with an average spacing of ~ 90 km, whereas only two in Chugoku (~ 500 km spacing). It may be thus confirmed that the contrasting volcano spacing in the SW Japan arc during Holocene has been continued from 2.6 Ma.

There certainly is a gap in Holocene volcanism along the volcanic front in central to southern Kyushu Island (Fig. 1). Analyses based on receiver function^33^ suggested that this volcanic gap may be caused by migration of slab-derived fluids back to the forearc mantle wedge along the surface of the slab to form low-velocity, possibly serpentinized mantle. As indicated in Fig. 2, however, the volcanic gap may not be so clearly observed when reararc volcanos are included, suggesting the contribution of slab-derived fluids to arc magmatism even in this region. Further detailed analyses may be required for better understanding the cause of the volcanic gap along the volcanic front of central Kyushu.

Figure 2 together with the above considerations then confirm that a larger number of volcanoes and the greater volume of mantle-derived magmas have formed in the Kyushu segment than in the Chugoku region. This observation may intuitively lead to the conclusion that the older and cooler PHS plate to the west of the KPR (> 50 my) is being subducted beneath the Kyushu region, whereas the younger and warmer PHS plate beneath the Chugoku region releases the water at shallow depths and cannot cause effective magma production in the mantle wedge. However, this simple mechanism could not be applied, since the boundary between the older and younger PHS plate, i.e., the KPR is currently located beneath the southern part of Kyushu Island (Fig. 1).

### Paleo-position of Kyushu-Palau Ridge (KPR): contrasting age of subducting Philippine sea plate

**Figure 3:**
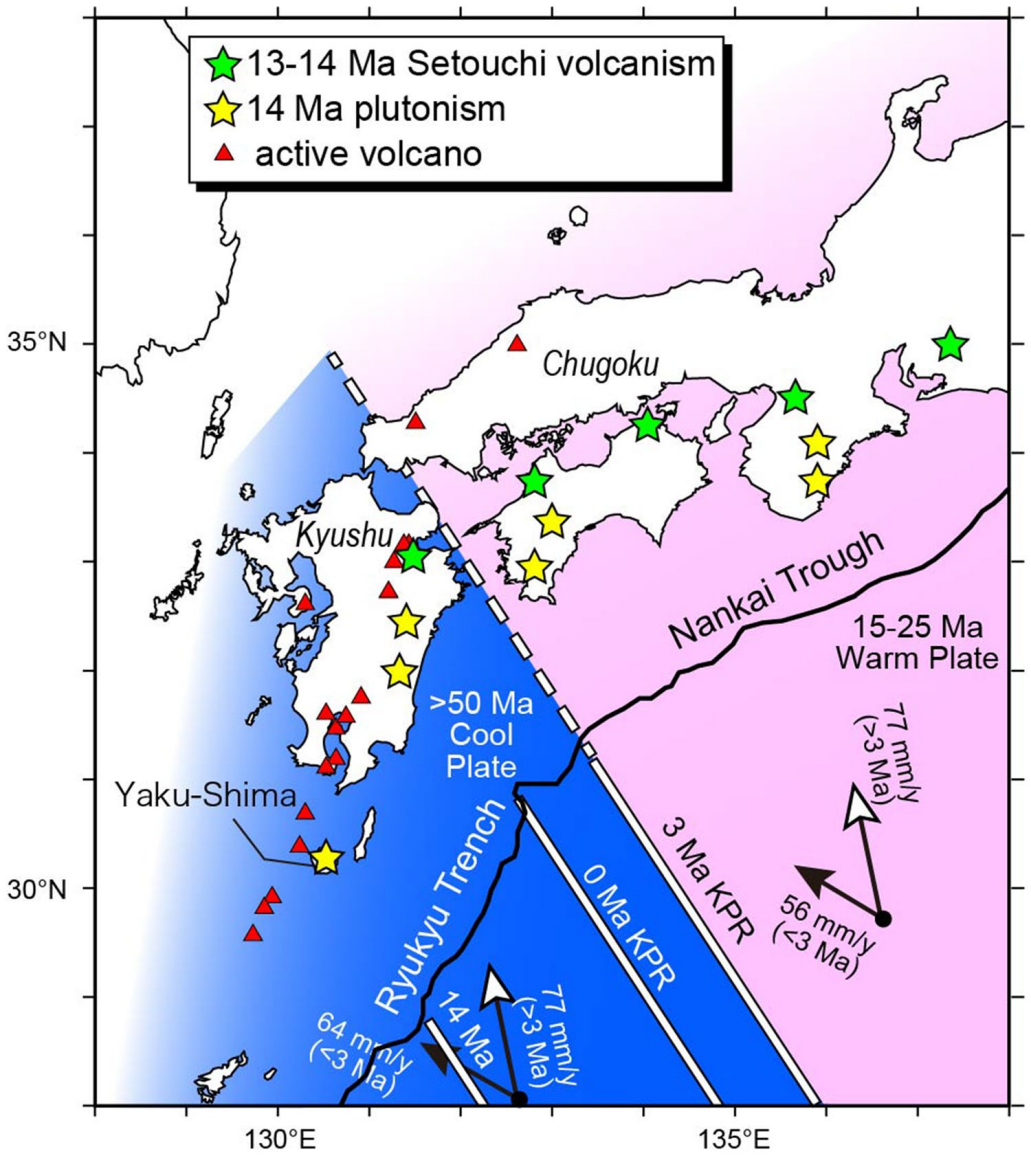
Positions of the Kyushu-Palau Ridge (KPR) at present, 3, and 14 Ma arranged by subduction of the PHS plate shown by arrows. Red triangles, active volcanoes; green and yellow stars, Setouchi and near trench felsic volcano-plutonic complexes occurred 13–14 Ma. The cool and warm lithospheres bordered by the KPR underlay Kyushu and Chugoku at 3 Ma, respectively.

The northern tip of the KPR, a remnant conjugate arc of the active Izu-Bonin-Mariana arc system, is located presently at the junction of the Nankai Trough and the Ryukyu Trench and is sinking beneath Kyushu Island (Figs. 1 and 3). The KPR plays a key role in the volcano-tectonic evolution of the SW Japan arc, as this forms a boundary between a younger (< 25 Ma) and an older (> 50 Ma) oceanic lithosphere and is composed of buoyant arc crust with the middle crust exhibiting seismic velocity similar to that of the bulk continental crust^34^.

Although it has been accepted generally that the subduction direction of the PHS plate changed from NNW to NW^35^, the timing of this change has been controversial. Geological and structural evolution of strata deposited in the forearc basin of at the eastern margin of the PHS subduction system, however, has led to the conclusion that it took place at 3 Ma and caused the stress change both in the NE and SW Japan arcs^36, 37^. If so, then the paleo-position of the KPR at 3 Ma could be reconstructed based on the current motion of the PHS plate (Fig. 3); the KPR was situated beneath the northeastern edge of Kyushu Island at 3 Ma. It is thus suggested that a > 50 Ma cool plate has distributed beneath the Kyushu region, whereas a warm lithosphere of the Shikoku Basin beneath the Chugoku region in SW Japan.

Characteristic volcanic rocks including mantle-derived high-Mg andesites erupted sporadically at 13–14 Ma and formed the Setouchi volcanic belt in the present forearc (Fig. 3), extending for **∼ **600 km with five major volcanic regions^38^. Synchronously with this magmatism, formed felsic volcano-plutonic complexes at 14 Ma^39, 40^ in the near-trench region of SW Japan (Fig. 3). If the dip angle of subduction has been unchanged for the last 14 my, the slab depth beneath these forearc or near trench magmatic belts would have been < 50 km, much shallower than that beneath most arc volcanic chains (110–170 km)^4^. Magma generation above such a shallow slab would require some additional conditions such as unusually high temperatures in the sinking lithosphere that could trigger slab melting and subsequent melt-mantle interaction^41^. This mechanism would be consistent with subduction of a newly-born lithosphere of the Shikoku Basin enforced by southward migration of the SW Japan arc sliver in association with opening of the Japan Sea^41^. If so, then the distribution of 13–14 Ma forearc magmatism in SW Japan could provide a constraint on the location of the KPR at that time; to the south of Yaku-Shima Island (Fig. 3). The rate of northward subduction of the PHS from 14 to 3 Ma can be then calculated as 77 mm/years, almost identical to the previous estimation^42^.

### Thermal structure and dehydration of the subducted PHS plate: contributions to arc magmatism

To understand migration of aqueous fluids associated with subduction of the young vs. old PHS plates and its role in causing contrasting volcano spacing in the Kyushu and Chugoku regions of the SW Japan arc, the thermal structures beneath two regions were estimated by 2D thermal structure models (Figs. S1, S4 and S6). Two end-member models are here constructed: One is a simple model (MODEL I) with constant slab age and subduction velocity (Figs. S2 and S3), and the other is a rather complex and positively close-to-reality model (MODEL II) that takes into account of the history of the subducted Philippine Sea plate and fits heat flow data best (Figs. S2, S3 and S5). In comparison to 2D models for these regions reported so far^43–47^, our thermal modeling has the following two advantages: (1) various heat sources were considered in the energy equation and (2) highly densely distributed heat flow data were used to constrain thermal structures and to estimate optimal values of model parameters in MODEL II (Fig. S5). Details of modeling are described in “Methods” and Supplementary Information (SI). Although 3D thermal modeling may provide new insights into the thermal evolution of a tectonically complex region such as SW Japan^48, 49^, we here adopted 2D, rather than 3D thermal models, as several hundreds of different values of model parameters with high spatial resolution must be tested to obtain high-resolution thermal structure suitable for examining the behavior of water in the subducted slab and the mantle wedge.

The calculated pressure–temperature (P–T) paths near to the surface of the PHS plate along the two profiles by MODEL I are shown in Fig. S4, together with H_2_O contents in the subducted oceanic crust and the downgoing peridotite under H_2_O-excess conditions calculated by Perple_X^50^ for a Shikoku Basin basalt and a peridotite (Table S2). It is indicated that temperatures in the two regions increase remarkably at a depth of ~ 40 km because this model does not incorporate a possible decoupling depth. As discussed later, an important point is that temperature along profile B (Chugoku) is much higher than that along profile A (Kyushu) mostly due to the age difference between them.

In MODEL II, we examined two possibly most preferable models among several hundreds of models with different values of model parameters in terms of the least square sense of the observed heat flow data along profiles A and B (Fig. S5b,c): One is the cold forearc model (MODEL II-1) incorporating decoupling to depths of 60 ~ 70 km, which has been suggested by previous works^43, 44, 47^, and the other is the hot mantle wedge corner model (MODEL II-2), which exhibits a remarkable temperature increase around a depth of 40 km along the plate interface as obtained by MODEL-I (Fig. S4). However, it is difficult to identify which model is better, because the difference in heat flow calculated for these two models is small and these heat flow values are largely consistent with the observed heat flow data. Further considerations on these two models are presented in the section “Possible decoupling depths” in SI. The estimated most suitable values of the model parameters for the cold forearc model along the two profiles are tabulated in Table S1.

Although three models, MODELs I, II-1, and II-2, provide different P–T profiles along the subducting crust (Fig. S4), all these models suggest commonly that slab temperatures along profile B is much higher than that along profile A mostly due to the age difference in the two regions. The behavior of H_2_O along with subduction of the PHS plate shall thus be examined based on the positively close-to-reality end-member model (MODEL II-1). It should be stressed, on the other hand, that discussions based on other models (MODELs I and II-2) reach to the conclusion on the cause of the contrasting volcano spacing in the Kyushu and Chugoku regions exactly the same as that based on the MODEL II-1.

**Figure 4:**
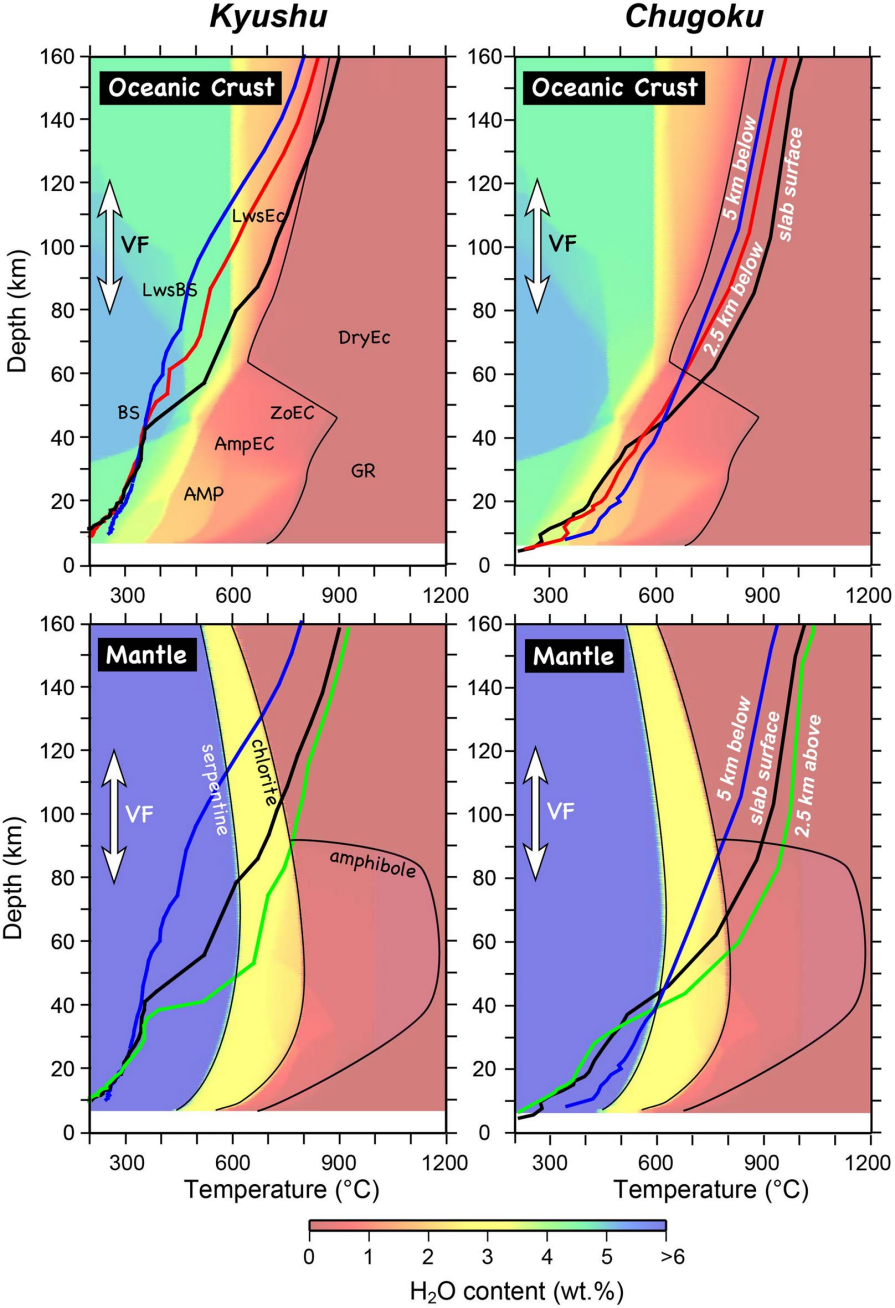
The calculated temperature distributions at the surface of the PHS plate along the profiles A and B passing through Kyushu and Chugoku districts, respectively for MODEL II-1 (Fig. 1). The metamorphic facies for the basaltic system, the stability limits of hydrous phases in the peridotite system, i.e., serpentine, chlorite, and amphibole, and H_2_O contents are also shown. *GS* green schist, *EA* epidote amphibolite, *BS* blue schist, *AMP* amphibolite, *GR* granulite, *AmpEC* amphibole eclogite, *ZoEC* zoisite eclogite, *LwsEC* lawsonite eclogite, *DryEC* dry eclogite.

**Figure 5:**
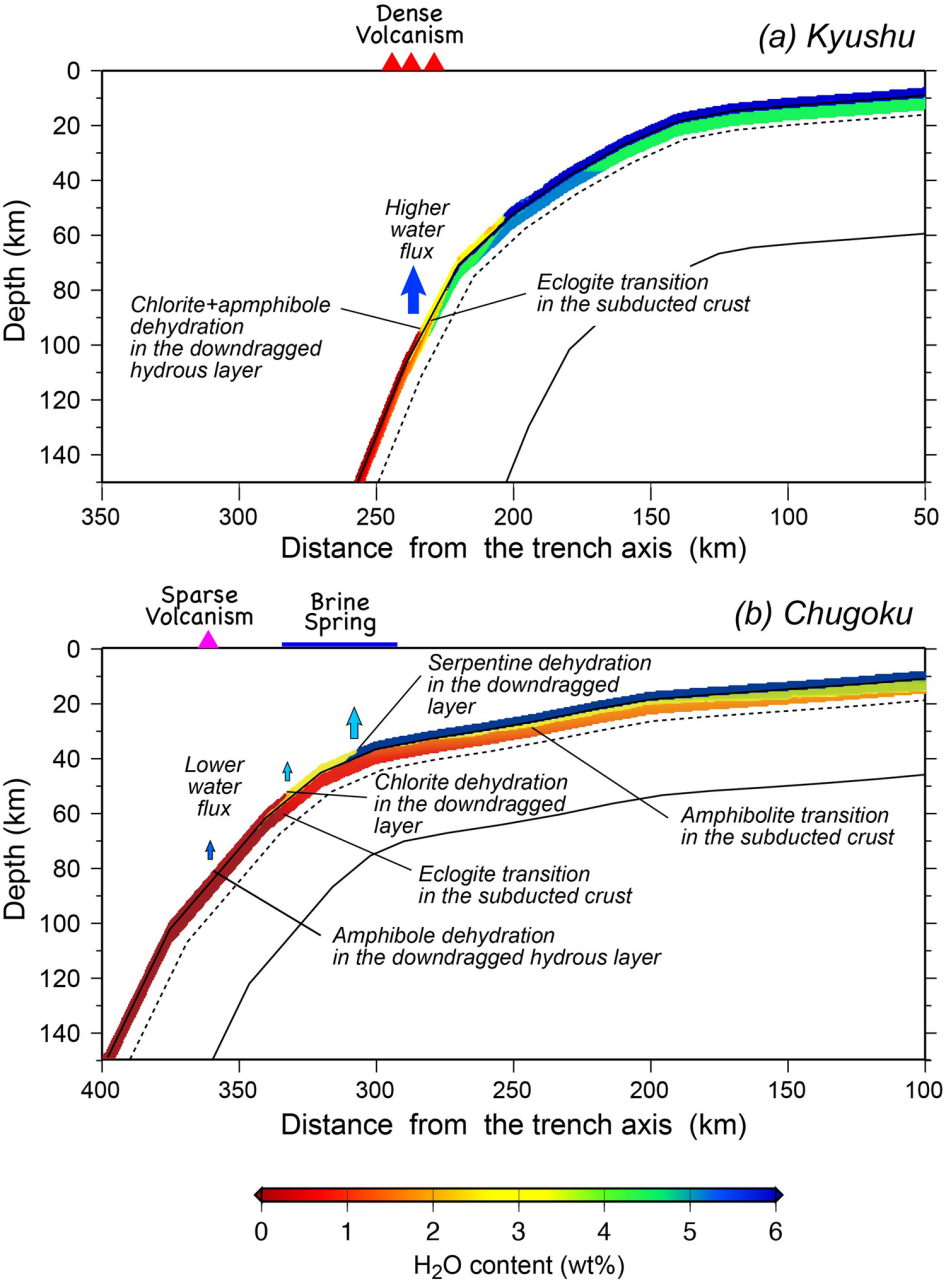
Dehydration and fluid release taking place along the subducted PHS plate beneath Kyushu (**a**) and Chugoku (**b**) for MODEL II-1. Higher water flux beneath Kyushu contributes to dense volcanism, while lower water flux caused by major dehydration reactions at a depth of ~ 50 km beneath Shikoku could not produce magmas actively to build a sparely-distributed volcanoes.

The present result for the Chugoku profile confirms the previous suggestion^9, 43^ that the oceanic crust sinking beneath this region is warm and most of the water in the oceanic crust is driven off at shallow depths not to trigger partial melting of the mantle wedge directly and may further provide insights into migration of fluids and its role in characteristic fluid-related activities in this subduction zone. One is the occurrence of tectonic tremors and LFEs^17, 18, 43, 51, 52^ taking place at ~ 30 km depths (Fig. 1). The major dehydration reaction in the subducted PHS oceanic crust corresponding to the transition to amphibolite facies takes place 20–40 km depths (Figs. 4 and 5) and triggers characteristic tectonic tremors and LFEs. The other distinctive subduction-related fluid activity in this region is the occurrence of deep-seated fluids exhibiting characteristic chemistry and often high temperature. Water discharged from the PHS crust migrates upwards to form hydrous peridotites at the base of the forearc mantle wedge, in which serpentine, chlorite and pargasitic amphibole may crystallize as major hydrous phases (Figs. 4 and 5). The hydrous peridotites are likely to be dragged downwards on the slab as a consequence of subduction of a rigid oceanic lithosphere into the viscous mantle, to supply aqueous fluids to the overlying dry mantle wedge^6^. Figure 4 also demonstrates that dehydration of serpentine and chlorite at the base of the hydrous peridotite layer occur, i.e., immediately above the slab surface, at ~ 40 km to release large amounts of water, which would be the source of characteristic deep-seated fluids referred to as the Arima-type brines (Figs. 1 and 5) as advocated geochemically^21–23, 41^.

Amphibole is then a hydrous phase in the down-dragged hydrous layer on the PHS plate that could transport water to deeper levels and decompose at a depth of ~ 90 km to release aqueous fluids (Fig. 4). This depth corresponds to the top of the PHS plate immediately beneath the active Sanbe volcano and other Quaternary volcanoes (Fig. 1). The Sanbe volcano is known by the occurrence of adakites exhibiting anomalously high Sr/Y ratios, leading to the conclusion that partial melting of the eclogitic PHS crust to cause magmatism^9, 53^. However, geochemical examination of adakites from this volcano and the surrounding suggests that these adakitic magmas are produced by melting of the lower crust, not of the subducted oceanic crust, in the presence of garnet, plagioclase, and amphibole^54–56^. It should be thus stressed that the amount of water supplied through amphibole dehydration in the down-dragged hydrous layer to cause arc magma production is much smaller than that in the original hydrous peridotites including serpentine and chlorite.

In contrast to the Chugoku region of SW Japan, the subducted PHS crust beneath the Kyushu region is much older (Fig. 3) and hence much cooler (Fig. 4). Providing that near the surface of the oceanic crust is significantly hydrated, then the subducting slab may largely dehydrate at 50 ~ 80 km depths (Fig. 4). In contrast, the hydrous layer at the base of the mantle wedge, i.e., immediately above the slab surface, enables to transport of a large amount of water to deep levels; chlorite and amphibole decompose to release water at depths of ~ 100 km (Fig. 4), causing much higher rates of melt production and consequently building more densely distributed volcanoes than a warmer thermal regime such as the Chugoku profile (Fig. 5).

It has been observed that hydration of mantle portion of the oceanic plate may occur at least in some outer-rise regions through bending-related faulting prior to subduction^57, 58^. The Pacific plate being subducted at the Japan Trench, for example, the presence of ~ 2 wt% H_2_O in the uppermost mantle immediately below the Moho could account for the observed seismic velocity reduction^58^. If this is the case for the subducting PHS plate, then slab-mantle-derived water may contribute further to magma generation in both the Kyushu and Chugoku regions as serpentine or chlorite decomposes to release H_2_O beneath the volcanic chains (mantle temperature profiles at 5 km below slab surface in Fig. 4). Even if this is the case, then the contribution of serpentine dehydration to magma generation may be inferred beneath the Kyushu, not Chugoku region.

The PHS plate is sinking normally along the Ryukyu Trench at the rate of > 63 mm/years, whereas obliquely along the Nankai Trough at the rate of 61 mm/years with a substantial trench-normal component of ~ 55 mm/years. The effective rate of subduction of the PHS plate is higher in the Kyushu than Chugoku regions. This may enhance the contrasting volcano distribution along the SW Japan arc, as higher rates of subduction tends to cause higher rates of magma production in the mantle wedge^3–5^.

## Concluding remarks

Quaternary volcanoes are distributed much more densely in the Kyushu than in the Chugoku segment along the SW Japan arc, although the PHS plate is currently being subducted beneath this arc. Tectonic reconstruction of the PHS plate that changed its direction of motion from NNE to NW at 3 Ma suggests an older (> 50 Ma) portion of the PHS oceanic crust with high dip angle has downgone beneath the Kyushu region, whereas the young (25–15 Ma) lithosphere of the Shikoku Basin with low dip angle in the forearc region of the Chugoku segment of the SW Japan arc. Geothermal calculations of the temperature distribution along the subducting PHS plate with different ages, together with petrological constraints on dehydration reactions taking place within both the downgoing crust and the overlying mantle wedge, demonstrate that much larger amounts of fluids are supplied to the magma source region beneath the Kyushu than the Chugoku regions, causing much higher density in volcano distribution in Kyushu. Water that are released from the young PHS plate beneath the forearc of the Chugoku region may cause characteristic tectonic tremors and LFEs, and be the source of brain springs.

## Methods

The calculation of 2D thermal structures in this study follows the previous models^47, 51, 52^. The momentum and energy equations were solved as a coupled problem, using the finite difference method. The model is a time-dependent, and considered possible heat sources such as viscous dissipation, adiabatic compression, frictional heating on the plate interface and temperature change caused by erosion and sedimentation during the Quaternary period in the energy equation. Viscosity is represented by a composite of diffusion creep and dislocation creep^59^, and the density depends solely on the temperature.

The present model setting is shown schematically in Fig. S1. The 2D box-type model has a horizontal length of 800 km and a depth of 400 km. Both the upper and lower crusts were set as conductive layers with respective thickness of 16 km. The accretionary prism was also incorporated into the model as a conductive layer. The thickness of the PHS plate at the Nankai Trough at the right model boundary is given based on the equation related to the age of the ocean floor^60^. The initial flow and temperature conditions for the model include no mantle flow and the half-space cooling with adiabatic compression at depths deeper than 50 km. As the boundary condition for flow fields, the normal stress is set to zero for the left, right, and bottom boundaries. As the boundary condition for temperature field, the model surface is set to 0 °C. Adiabatic conditions are assumed for the left and bottom boundaries. Geometry models of the PHS slab were taken^14, 15, 61^. The PHS plate is assumed to subduct along a prescribed guide^62^ whose length gradually extends from the right boundary from 14 Ma. Grid sizes for stream functions and temperatures are 4 $\times$ 4 km and 2 $\times$ 2 km, respectively, and the stream function is evaluated at the same grid spacing as the temperature field via the third-order Spline interpolation. Remeshing with 1 km for the mantle wedge corner, where intense flow is expected to occur, is performed at each time step^63, 64^.

For a simple model (MODEL I), we gave constant age of 50 my and constant velocity of 64 mm/years for the subducting PHS plate along profile A, whereas those of 17 my and 44 mm/years along profile B throughout the calculated period of 14 my (Figs. S2 and S3). We did not use heat flow data, and a decoupling depth is not incorporated into the model.

On the other hand, for a rather complex and positively close-to-reality model (MODELs II-1 and II-2), the depth and age dependent temperature distribution determined by the plate cooling model RT1^65^ is imposed at the right boundary. Time-dependent age and subduction velocity along profiles A and B were given, following the assumed subduction history (Figs. 1, 4, S2 and S3). For MODELs II-1 and II-2, we also used heat flow data from high-quality high-density Hi-net borehole and BSRs, which have not been used except for studies of our group^46, 47, 51, 52^, in addition to previously-used land boreholes and marine heat probes, resulting in the high-density heat flow data (Fig. S5a) to constrain the thermal structures along the profiles passing through Kyushu and Chugoku regions. This enables us to estimate thermal structures with high spatial resolution from shallow to deeper portions in association with subduction of the PHS plate. We correctly picked up only data along the two profiles within one-sided width of 30 km (Fig. S5b,c), Tables S2 and S4), and estimated optimal thermal models in which the calculated heat flow fits best with the observed values by least square method. It should be noted that spatial distributions of the observed densely-distributed heat flow along the two profiles obtained in this study are rather different from those of previous studies^44^; Shorter wavelength patterns can be identified, which should be explained by introducing heat sources such as temperature change caused by erosion and sedimentation during the Quaternary period^46^. To better reproduce the observed heat flow data along the two profiles, pore pressure ratio on the plate interface, radioactive heating per unit volume in the accretionary prism, initial age of the PHS plate at the Nankai Trough, age discontinuity passing through the KPR, depth range and thickness of a low-viscosity layer attached on the plate interface, and its viscosity contrast against the surrounding region are assumed to be unknown free parameters (Table S1). Then, we performed grid search for several hundreds of different values of such free parameters for the respective profiles. Other details of the thermal modeling are described elsewhere^47, 51, 52^.

## Supplementary information

Supplementary Information.

## Data Availability

All data generated and analyzed in this study are included in main text or Supplementary Information.
