# Supplementary material for: Contrasting volcano spacing along SW Japan arc caused by difference in age of subducting lithosphere
Source: Sci Rep. 2020 Sep 14;10:15005. doi: 10.1038/s41598-020-72173-6 (PMC7490715; doi:10.1038/s41598-020-72173-6)
Supplement: Supplementary file 1 — Supplementary Information. [file 41598_2020_72173_MOESM1_ESM.docx]

Supplementary information

Contrasting volcano spacing along SW Japan arc caused by difference in age of subducting lithosphere

Yoshiyuki Tatsumi*^1,2^, Nobuaki Suenaga^3^, Shoichi Yoshioka^3,2^, Katsuya Kaneko^2,1^ and Takumi Matsumoto^4^

^1^ Kobe Ocean-Bottom Exploration Center, Kobe University, Kobe 658-0022, Japan

^2^ Department of Planetology, Kobe University, Kobe 657-8501, Japan

^3^ Research Center for Urban Safety and Security, Kobe University, Kobe 657-8501, Japan

^4^ National Research Institute for Earth Science and Disaster Resilience, Tsukuba 305-0006, Japan

*Correspondence author: [tatsumi@diamond.kobe-u.ac.jp](mailto:tatsumi@diamond.kobe-u.ac.jp)

**Contents**

Sensitivity tests

Comparison with our previous studies

Possible decoupling depths

References

Tables S1-S5

Figures S1-S10 and their captions

**Sensitivity tests**

To investigate parameter dependency on the results of the thermal modeling, we carried out sensitivity tests, by changing the most suitable values of the model parameters (MODEL II-1) along the profile A-A’: We changed values of respective model parameters within maximum possible range for 7 CASEs, and checked their effects on heat flow and pressure-temperature (p-T) paths. Therefore, it should be noted that they are extreme CASEs.

When the convergence rate during the period from 14 to 3 Ma became half (CASE 1 in Table S5), calculated heat flow increased up to 9.1 mW/m^2^ within horizontal range from 60 to 190 km (Fig. S7), resulting in temperature increase of ~100°C in the p-T path (Fig. S9). The effect of halving the convergence rate during the period from 3 to 0 Ma (CASE 2 in Table S5) was little, only producing heat flow increase by 5.2 mW/m^2^ within horizontal range from 100 to 170 km (Fig. S7). When we changed seafloor age difference across the KPR from 30 to 10 Myr (CASE 3-1 in Table S5), large increase of heat flow, reaching 27.4 mW/m^2^ at the trough axis, was identified in the forearc region at the horizontal distance shorter than 170 km (Fig. S7), resulting in larger and smaller temperature differences at shallower (~80 km ) and deeper regions, respectively (Fig. S9). Oppositely, by increasing the value from 30 to 50 Myr (CASE 3-2 in Table S5), large decrease in heat flow was expected, reaching 14.5 mW/m^2^ in the similar horizontal range (Fig. S7), yielding colder temperature at a depth shallower than 45 km (Fig. S9). Regarding down-dip depth of the low-viscosity layer, comparison with the model without incorporating the layer (MODEL II-2; CASE 4-1 in Table S5) produced increase in heat flow up to 6.0 mW/m^2^ in the horizontal range from 170 to 260 km (Fig. S8), but temperature difference was large at depths deeper than 40 km (Fig. S10). When we deepened the decoupling depth from 70 to 100 km (CASE 4-2 in Table S5), the difference in heat flow was negligible (Fig. S8). These points will be discussed in more detail in the section of “Possible decoupling depths”. Finally, when we changed pore pressure ratio from 0.98 to 1.00 (CASE 5 in Table S5), heat flow decreased down to 10.7 mW/m^2^ in the horizontal range from 20 to 190 km (Fig. S8), producing large temperature decrease at depths shallower than a depth of about 45 km (Fig. S10).

The results of these sensitivity tests for the extreme cases indicate that heat flow and p-Tpaths are most sensitive to the age difference across the KPR, which is consistent with our hypothesis, followed by pore pressure ratio and convergence rate. Difference in decoupling depths is the most insensitive parameter in heat flow among them. Therefore, it is essential to estimate subduction history and pore pressure ratio correctly to draw a reliable conclusion in our study.

**Comparison with our previous studies**

In this study we newly proposed subduction history during the period from 14 Ma to 3 Ma, namely, northward motion of the PHS plate with velocity of 77 mm/yr (Fig. 3). On the other hand, clockwise rotation of the PHS plate was considered in our previous studies^1,2^ based on geological study^3^. We also extended the pick-up width of heat flow data of one-sided width of 30 km in this study, whereas that of 20 km was taken in our previous studies^1,2^. Furthermore, we did not mention basaltic volcanism in our previous studies^1,2^, and used phase diagrams of hydrous minerals are different: In this study we used phase diagrams using Perple_X^4^, whereas we used those of MORB^5^ and slab mantle^6^ in our previous studies^1,2^.

Although the profile A-A’ in this study is located closely to the profile in our previous study^1^, the former is located northward slightly as compared with the latter^1^. Therefore, pick-up heat flow data were slightly different between the two profiles. As a result of numerical simulations, the most suitable thermal model including a decoupling depth to minimize root-mean-square between observed and calculated heat flows in this study was similar to the model in our previous study^1^: A model with a thin low viscosity layer to a depth of 70 km is the most preferable. Difference in adopted subduction histories little affected difference in the two thermal models.

The profile B-B’ in this study is located closely to ’the profile A’ in our previous study^2^, in which temperature in the mantle wedge is higher than that obtained in this study. The difference of temperatures between these two models is mainly caused by the difference in dip angle, pore pressure ratio, subduction history, and incorporation of a decoupling depth. Dip angle in our previous study^2^ is larger than that in this study because the former profile passes through the Kii Peninsula, which is located east of Shikoku. When the dip angle becomes larger, hot mantle flow tends to flow into the mantle wedge corner more intensely, resulting in higher temperature there, and vice versa. The most suitable values of pore pressure ratio in the present and previous^2^ studies are 1.00 and 0.97, respectively. The larger the value is, the lower the temperature is. Because of the difference in adopted subduction histories, convergence rate in the across-arc direction was dominant in this study, whereas that in the along-arc direction was dominant in our previous study^2^ during the period from 11 Ma to 7 Ma, yielding lower temperature in the former than the latter. Incidentally, this effect affects more seriously along profile B-B’ than profile A-A’ in this study because the former is situated almost perpendicular to the trough axis. Because of these three factors, temperature at the mantle wedge corner along the profile B-B’ obtained in the hot mantle wedge corner model in this study (MODEL II-2) became approximately 120°C lower than that obtained in our previous study^2^. Furthermore, in MODEL II-1, we incorporated a decoupling depth whose most suitable value was estimated as 60 km along the profile B-B’, whereas a decoupling depth was not considered in our previous model^2^. Incorporation of a decoupling depth produces a colder mantle wedge. As a result, temperature at the mantle wedge corner for MODEL II-1 became lower than that for MODEL II-2, resulting in temperature difference reaching 280°C there between MODEL II-1 and our previous study^2^.

**Possible decoupling depths**

Whether forearc is cold or not has been evaluated using heat flow data. Previous study^7^ used the concept of partial decoupling to decoupling depths of 70-90 km^8^. However, the previous study^8^ used heat flow data obtained from previously-used marine heat probe and land borehole, and plotted them by collecting located even far from the two profiles passing through Chugoku and Kyushu due to shortage of heat flow data along the exact profiles. The calculated heat flows assuming a cold forearc model in the previous study^8^ do not explain observed heat flow data, especially along the profile passing through Kyushu. It indicates that a decoupling depth to depths of about 70-90 km is not an approved fact, but a hypothesis. In this study we used heat flow data from Hi-net borehole and BSRs, which have not been used except studies of our group^1, 2, 9, 10^, in addition to the above-described heat flow data, realizing the much higher density, higher quality heat flow data (Fig. S5(a)). We correctly picked up only data along the two profiles within one-sided width of 30 km (Figs. S5(b) and (c)), and estimated most preferable model by grid search, testing several hundreds of different models including the cold forearc models. As a result, we found that the cold forearc model (MODEL II-1) and the hot mantle wedge corner model (MODEL II-2) cannot be distinguished by their expected heat flows to the horizontal distance of approximately 280 km along profile B-B’. This is because the dip angle of the slab along profile B-B’ is low (~10°) to a horizontal range of about 280 km, and so, mantle flow does not flow into the corner of the mantle wedge effectively because of the effect of the shallow dip angle, even for the non-decoupling hot mantle wedge corner model (MODEL II-2). More importantly, from Figs. S5(b) and (c), it would be impossible to determine whether the cold forearc model (MODEL II-1) or the hot mantle wedge corner model (MODEL II-2) is preferable because difference in calculated heat flows for the two models is not so large within short horizontal range along both profiles A-A’ and B-B’, and the heat flow data are scattered there as well. In fact, the maximum differences in heat flow between the two models are 6.0 mW/m^2^ and 8.8 mW/m^2^ along profiles A-A’ and B-B’, respectively. This slight difference in calculated heat flows within short horizontal range originates from high dip angles of the slab near the mantle wedge corners. Therefore, such a cold forearc model is not necessarily supported even if we employed the high-density high-quality heat flow data. Therefore, it would be premature to conclude that decoupling depths of all the global subduction zones are 70-90 km, and they should be re-evaluated more carefully.

**References**

1. Suenaga, N., Yoshioka, S., Matsumoto, T. & Ji, Y. Two-dimensional thermal modeling associated with subduction of the Philippine Sea plate in southern Kyushu, Japan, *Tectonophysics* **723**, 288-296 (2018).
2. Suenaga, N., Yoshioka, S., Matsumoto, T., Manea, V. C., Manea, M, & Ji, Y. Two-dimensional thermal modeling of the Philippine Sea plate subduction in central Japan: Implications for gap of low-frequency earthquakes and tectonic tremors, *J. Geophys. Res.* *Solid Earth* **124**, 6848-6865 (2019).
3. Kimura, G., Hashimoto, Y., Kitamura, Y., Yamaguchi, A., & Koge, H. (2014). Middle Miocene swift migration of the TTT triple junction and rapid 4 crustal growth in SW Japan. *Tectonics*, **33**, 1219–1238. https://doi.org/10.1002/2014TC003531.
4. Connolly, J. A. D. Computation of phase equilibria by linear programming: A tool for geodynamic modeling and its application to subduction zone decarbonation. *Earth Planet. Sci. Lett.,* **236**, 524–541 (2005).
5. Omori, S., Kita, S., Maruyama, S. & Santosh, M. Pressure-temperature conditions of ongoing regional metamorphism beneath the Japanese Islands. *Gondwana Res.* **16**, 458–469 (2009).
6. Hacker, B., Abers, G. & Peacock, S. Subduction factory 1. Theoretical mineralogy, densities, seismic wave speeds, and H_2_O contents. *J. Geophys. Res.* **108**, <http://dx>. doi.org/10.1029/2001JB001127 (2003).
7. Syracuse, E. M., van Keken, P. E. & Abers, G. A. The global range of subduction zone thermal models, *Phys. Earth Planet. Inter.* **183**, 73–90 (2010).
8. Wada, I. & Wang, K. Common depth of slab-mantle decoupling: Reconciling diversity and uniformity of subduction zones, *Geochem. Geophys. Geosys.* **10**, Q10009; 10.1029/2009GC002570 (2009).
9. Yoshioka, S., Suminokura, Y., Matsumoto, T. & Nakajima, J. Two-dimensional thermal modeling of subduction of the Philippine Sea plate beneath southwest Japan, *Tectonophys.* **608**, 1094-1108 (2013).
10. Suenaga, N., Yoshioka, S. & Matsumoto, T. Relationships among temperature, dehydration of the subducting Philippine Sea plate, and the occurrence of a megathrust earthquake, low-frequency earthquakes, and a slow slip event in the Tokai district, central Japan. *Phys. Earth Planet. Int.* **260**, 44-52 (2016).

**Table S1 Parameter setting for the best fit model (MODEL II-1) along the profiles A-A’ and B-B’**

| Model parameter | | Profile A-A’ | Profile B-B’ |
| --- | --- | --- | --- |
| Convergence rate (14Ma-3Ma) along the profile (fixed) | | 5.52 $\left[\text{cm/yr}\right]$ | 7.09 $\left[\text{cm/yr}\right]$ |
| Convergence rate (3Ma-0Ma) along the profile* (fixed) | | 6.40 $\left[\text{cm/yr}\right]$ | 5.50 $\left[\text{cm/yr}\right]$ |
| Pore pressure ratio (1.00-pore pressure ratio) | | 0.98 (0.02) | 1.00 (0.00) |
| Seafloor age difference across the KPR | | 30 $\left[\text{Myr}\right]$ | ― |
| Age when the profile passes through the KPR (fixed) | | 4.5 $\left[\text{Ma}\right]$ | ― |
| Potential temperature (fixed) | | 1350 [℃]** | |
| Down-dip depth of the  low-viscosity layer | | 70 $\left[\text{km}\right]$ | 60 $\left[\text{km}\right]$ |
| Thickness of the low-viscosity layer | | 7 $\left[\text{km}\right]$ | 7 $\left[\text{km}\right]$ |
| Viscosity difference between the  low-viscosity layer and the surrounding mantle | | $1.0\times10^{-2}$ $\left[\text{times}\right]$ | $1.0\times10^{-2}$ $\left[\text{times}\right]$ |
| Density of the mantle  (fixed) | | 3400 $\left[\text{kg/m}^{3}\right]$*** | |
| Thickness of the continental crust (fixed) | | Upper crust :  16 $\left[\text{km}\right]$  Lower crust  16 $\left[\text{km}\right]$ | Upper crust :  16 $\left[\text{km}\right]$  Lower crust :  16 $\left[\text{km}\right]$ |
| Viscosity (fixed) | | Burkett and Billen (2010)**** | |
| Length of the accretionary prism (fixed) | | 200 $\left[\text{km}\right]$ | 174 $\left[\text{km}\right]$ |
| Radioactive heating per unit volume | accretionary prism | 3.0 $\left[\text{mW/m}^{2}\right]$ | 2.5 $\left[\text{mW/m}^{2}\right]$ |
|  | upper crust (fixed) | 1.9 $\left[\text{mW/m}^{2}\right]$ | |
| Thermal conductivity***** | accretionary prism | 2.9 $\left[\text{W/m}\cdot\text{K}\right]$ | |
|  | upper crust | 2.5 $\left[\text{W/m}\cdot\text{K}\right]$ | |
|  | lower crust | 2.5 $\left[\text{W/m}\cdot\text{K}\right]$ | |

* DeMets et al. (2010)

** Takenaka et al. (1999)

*** Yoshioka & Sanshadokoro (2002)

**** Burkett & Billen (2010)

***** Turcotte & Schubert (1982)

DeMets, C., Gordon, R. G., and Argus, D. F., 2010. Geologically current plate motions, Geophys. J. Int., 181, 1-80.

Takenaka, S., Sanshadokoro, H. & Yoshioka, S., 1999. Velocity anomalies and spatial distributions of physical properties in horizontally lying slabs beneath the Northwestern Pacific region. Phys. Earth Planet. Inter. 112, 137-157.

Yoshioka, S. & Sanshadokoro, H., 2002. Numerical simulations of the deformation and dynamics horizontally lying slabs. Geophys. J. Int. 151, 69-82.

Burkett, E.R. & Billen, M.I., 2010. Three-dimentionality of slab detachment due 　　　　 to ridge-trench collision: Laterally simultaneous boundinage versus tear propagation. Geochem. Geophys. Geosyst. 11, doi:10.1029/2010GC003286.

Turcotte, D. L. & Schubert, G., 1982. Geodynamics applications of continuum physics to geologic problems. John Wiley & Sons., 450 pp.

**Table S2** Compositions of basaltic crust and peridotite.

|  | Shikoku Basin Basalt** | Peridotite*** |
| --- | --- | --- |
| SiO_2_ | 51.9 | 45.4 |
| A_l2_O_3_ | 16. 5 | 4.7 |
| FeO* | 9.3 | 8.4 |
| MgO | 7.2 | 37.4 |
| CaO | 12.2 | 3.7 |
| Na_2_O | 2.9 | 0.39 |

* Total iron as FeO

** Dick et al. (1980)

*** Depleted MORB Mantle minus 6% Primary MORB (Workman and Hart, 2005)

Dick, H.J.B., Marsh N.G., and Bullen, T.D. (1980) Deep sea drilling project leg 58 abyssal basalts from the Shikoku Basin: their petrology and major-element geochemistry, Initial Reports of the Deap Sea Drilling Project, 58, 843-872.

Workman, R.K. and Hart, S.R. (2005) Major and trace element composition of thedepleted MORB mantle (DMM), Earth Planet. Sci. Lett., 231, 53-72.

**Table S3** Heat flow values of Hi-net observation wells along profile A-A’.

| Longitude (°E) | Latitude (°N) | Heat flow values*  (mW/m^2^) |
| --- | --- | --- |
| 130.5603 | 32.62 | 104.42 |
| 130.9069 | 32.4867 | 104.56 |
| 130.58 | 32.2883 | 88.65 |
| 130.7394 | 32.2019 | 132.85 |
| 130.9119 | 32.2175 | 48 |
| 130.7544 | 32.6311 | 72.24 |
| 131.1069 | 32.4719 | 68.58 |
| 131.2692 | 32.3436 | 100.37 |
| 131.4667 | 32.3572 | 98.25 |
| 131.1969 | 32.1953 | 83.87 |
| 131.5333 | 32.2097 | 21.41 |
| 131.0642 | 32.0386 | 44.72 |
| 131.2924 | 32.0181 | 39.58 |
| 131.4724 | 32.0173 | 33.7 |
| 129.7675 | 33.2089 | 109.68 |
| 129.8125 | 33.1222 | 59.16 |
| 129.8049 | 32.9519 | 74.48 |
| 130.1431 | 32.9069 | 137.12 |
| 129.8647 | 32.6965 | 98.24 |

*Matsumoto (2007)

Matsumoto, T., 2007. Terrestrial heat ﬂow anomaly at non-volcanic area in Southwest Japan based on the NIED Hi-net. Japan Geoscience Union Meeting T154-001 (in Japanese).

**Table S4** Heat flow values of Hi-net observation wells along profile B-B’.

| Longitude (°E) | Latitude (°N) | Heat flow  values*  (mW/m^2^) |
| --- | --- | --- |
| 133.0608 | 34.1889 | 61.17 |
| 133.0259 | 34.3704 | 46.37 |
| 132.9506 | 34.545 | 79.8 |
| 133.1392 | 34.5153 | 43.92 |
| 133.3539 | 34.3753 | 50.07 |
| 133.4206 | 34.5533 | 41.94 |
| 132.9144 | 34.9103 | 81.26 |
| 132.955 | 34.6983 | 67.42 |
| 133.1701 | 34.8193 | 57.04 |
| 132.6715 | 34.6597 | 91.28 |
| 132.7949 | 34.454 | 59.49 |
| 133.7107 | 34.1948 | 35.67 |
| 134.1228 | 33.5383 | 101.31 |
| 133.8269 | 33.6714 | 72.22 |
| 134.1629 | 33.2832 | 54.5 |
| 133.8218 | 33.5261 | 50 |
| 133.5786 | 33.751 | 38.97 |
| 132.7249 | 35.2218 | 107.72 |
| 132.5328 | 35.0886 | 87.45 |
| 132.6422 | 34.8661 | 91.95 |
| 132.4233 | 34.8772 | 67.47 |
| 132.8583 | 35.1603 | 115.68 |
| 132.3094 | 35.0056 | 75.86 |
| 133.8959 | 33.9964 | 77.38 |

*Matsumoto (2007)

Matsumoto, T., 2007. Terrestrial heat ﬂow anomaly at non-volcanic area in Southwest Japan based on the NIED Hi-net. Japan Geoscience Union Meeting T154-001 (in Japanese).

**Table S5** Model parameters for sensitivity test along profile A-A’.

| CASE | Model parameter | Best fit model | Sensitivity test | Maximum difference in heat flow for the best fit model subtracted from the respective case |
| --- | --- | --- | --- | --- |
| 1 | Convergence rate (14-3Ma) along the profile | 5.52 $\left[\text{cm/yr}\right]$ | 2.26 $\left[\text{cm/yr}\right]$ | 9.1 $\left[\text{mW/m}^{2}\right]$ |
| 2 | Convergence rate (3Ma-0Ma) along the profile | 6.40 $\left[\text{cm/yr}\right]$ | 3.20 $\left[\text{cm/yr}\right]$ | 5.2 $\left[\text{mW/m}^{2}\right]$ |
| 3-1 | Seafloor age difference across the KPR | 30 $\left[\text{Myr}\right]$ | 10 $\left[\text{Myr}\right]$ | 27.4 $\left[\text{mW/m}^{2}\right]$ |
| 3-2 |  |  | 50 $\left[\text{Myr}\right]$ | -14.5 $\left[\text{mW/m}^{2}\right]$ |
| 4-1 | Down-dip depth of the low-viscosity layer | 70 $\left[\text{km}\right]$ | No layer | 6.0 $\left[\text{mW/m}^{2}\right]$ |
| 4-2 |  |  | 100 $\left[\text{km}\right]$ | -0.2 $\left[\text{mW/m}^{2}\right]$ |
| 5 | Pore pressure ratio | 0.98 | 1.00 | -10.7 $\left[\text{mW/m}^{2}\right]$ |

**Figure S1** Schematic figure of the 2-D box-type thermal convection model used in this study. Red line on the plate interface denotes the region where we applied the frictional heating in the energy equation. Enclosed in brown denotes the area where we applied the low viscosity layer along profiles A-A’ and B-B’ passing through Kyushu and Chugoku, respectively. Black open arrows denote the subduction velocity, for which we gave along the prescribed guide. As a boundary condition for temperature and flow fields, adiabatic condition (left and bottom boundaries) and zero-normal stress condition (left, right, and bottom boundaries) are applied, respectively. Temperature at the model surface is fixed to be 0 °C. Constant depth-dependent temperature distribution and time- and age-dependent temperature distribution are imposed on the right boundary for MODEL I and MODELs II-1 & II-2, respectively.

(b)

(a)

**Figure S2** Subduction history of the subducting PHS plate along the profile A-A’ passing through Kyushu in Fig. 1 during the period from 14 Ma to the present (0Ma) for the very simple model (MODEL I) and the close-to-reality model (MODELs II-1 & II-2). In MODEL I, we assumed the constant values for the subduction velocity and the slab age from 14 Ma to the present. (a) Temporal change of the subducting velocity of the PHS plate. Red dashed line denotes MODEL I. Black line represents MODELs II-1 & II-2, for which subduction velocity at the Nankai Trough is assumed to be 77 mm/yr in the direction of N11°W during a period from 14 Ma to 3 Ma, and 64 mm/yr in the direction of N58.5°W during a period from 3 Ma to 0 Ma*. The subduction velocity is projected onto the profile A-A’ (N55.3°W). (b) Temporal change of the age of the PHS plate at the Nankai Trough. Red dashed and black lines represent MODEL I and MODELs II-1 & II-2, respectively.

* DeMets et al. (2010)

DeMets, C., Gordon, R. G., and Argus, D. F. (2010) Geologically current plate motions, Geophys. J. Int., 181, 1-80.

(a)

(b)

**Figure S3** Same as Figure S2 except for profile B-B’passing through Chugoku in Fig. 1. (a) Temporal change of the subducting velocity of the PHS plate. Red dashed line denotes MODEL I. Black line represents MODELs II-1 & II-2, for which subduction velocity at the Nankai Trough is assumed to be 77 mm/yr in the direction of N11°W during a period from 14 Ma to 3 Ma, and 60.6 mm/yr in the direction of N59°W during a period from 3 Ma to 0 Ma*. The subduction velocity is projected onto the profile B-B’ (N34.15°W). (b) Temporal change of the age of the PHS plate at the Nankai Trough. Red dashed and black lines represent MODEL I and MODELs II-1 & II-2, respectively.

* DeMets et al. (2010)

DeMets, C., Gordon, R. G., and Argus, D. F., 2010. Geologically current plate motions, Geophys. J. Int., 181, 1-80.

(c)

(b)

(a)

**
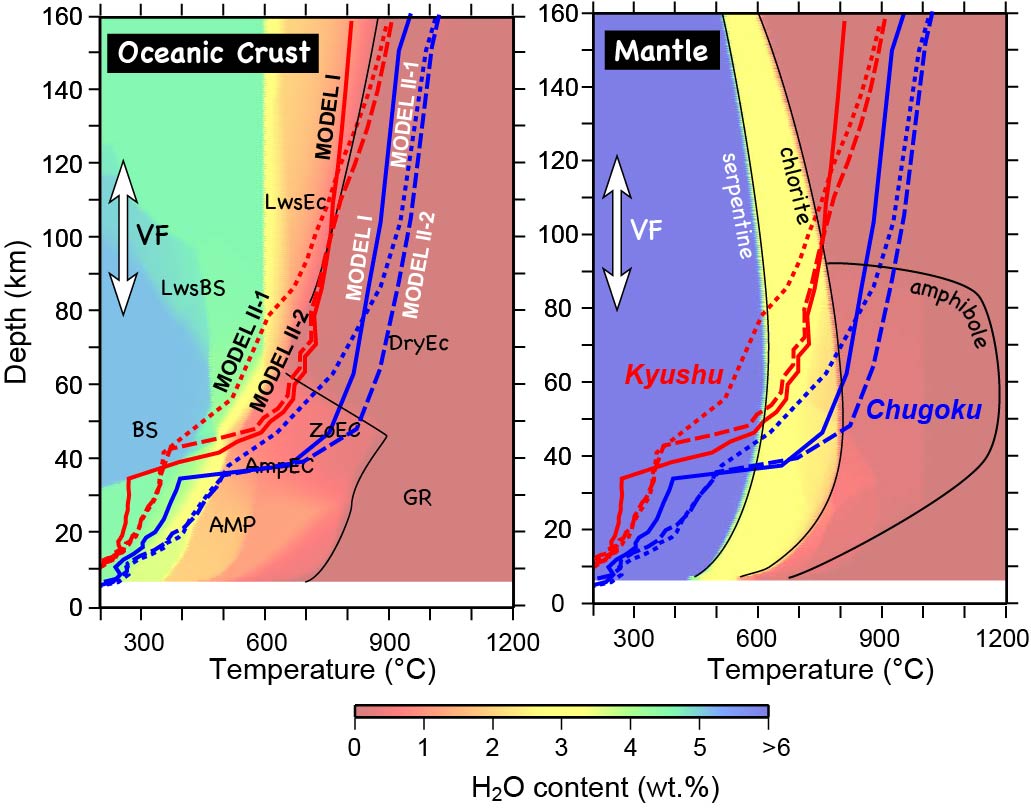
**

**Figure S4.** p-T paths (Figs. S2 & S3) along the upper surface of the subducting PHS plate plotted on the phase diagram of H_2_O content of MORB oceanic crust in (a) and mantle peridotite in (b) calculated from Perple_X*. Red and blue lines represent p-T paths at the upper surface of the PHS plate along the profiles A-A’ (Kyushu) and B-B’ (Chugoku), respectively. Solid, dotted, and dashed lines denote temperature-depth paths for MODELs I, II-1, and II-2, respectively. The metamorphic facies for the basaltic system and the stability limits of hydrous phases in the peridotite system, i.e., serpentine, chlorite, and amphibole, are also shown. GS, green schist; EA, epidote amphibolite; BS blue schist; AMP, amphibolite; GR, granulite; AmpEC, amphibole eclogite; ZoEC, zoisite eclogite; LwsEC. lawsonite eclogite; DryEC, dry eclogite.

* Connolly, J. A. D. Computation of phase equilibria by linear programming: A tool for geodynamic modeling and its application to subduction zone decarbonation. *Earth Planet. Sci. Lett.,* **236**, 524–541 (2005).

(b)

(a)

(c)

**Figure S5** (a) Spatial distribution of heat flow data in southwest Japan. Red circles, blue triangles, and pink squares denote the heat flow observed by BSRs*, land borehole and marine heat probe**, and Hi-net observation wells***, respectively. (b) Observed and calculated heat flows along the profile A-A’ in (a). Observed data within a one-sided width of 30 km along the profile are plotted with the same symbols as (a). The black solid line denotes the calculated heat flow for the suitable model (MODEL II-1), for which optimal values of 0.98, 3.0 mW/m^2^, 7 km, 70 km, and 30 Myr were adopted for pore pressure ratio, radioactive heating per unit volume in the accretionary prism, thickness of the low viscosity layer, down-dip depth of the low viscosity layer, and the age discontinuity passing through the KPR, respectively. Blue broken line denotes the calculated heat flow for the most suitable model without a low viscosity layer (MODEL II-2), whose other parameter setting is the same as that of MODEL II-1. (c) Same as (b) except for profile B-B’, and optimal values of 1.00, 2.5 mW/m^2^, 7 km, and 60 km were adopted for pore pressure ratio, radioactive heating per unit volume in the accretionary prism, thickness of the low viscosity layer, and down-dip depth of the low viscosity layer, respectively. Blue broken line denotes the calculated heat flow for the most suitable model without a low viscosity layer (MODEL II-2), whose other parameter setting is the same as that of MODEL II-1.

(c)

* Ashi et al. (2002)

** Tanaka et al. (2004); Yamano (2004)

*** Matsumoto (2007)

Ashi, J., Tokuyama, H., and Taira, A., 2002. Distribution of the methane hydrate BSRs and its implication for the prism growth in the Nankai Trough. Marine Geology 3110,1-15.

Tanaka, A., Yamano, M., Yano, Y., and Sasada, M. (2004). Geothermal gradient and heat flow data in and around Japan, digital geoscience map DGN P-5.

Yamano, M. (2004). Heat Flow Data in and around Japan, Digital Geoscience Map DGM P-5, Geological Survey of Japan.

Matsumoto, T., 2007. Terrestrial heat ﬂow anomaly at non-volcanic area in Southwest Japan based on the NIED Hi-net. Japan Geoscience Union Meeting T154-001 (in Japanese).

(a)

**
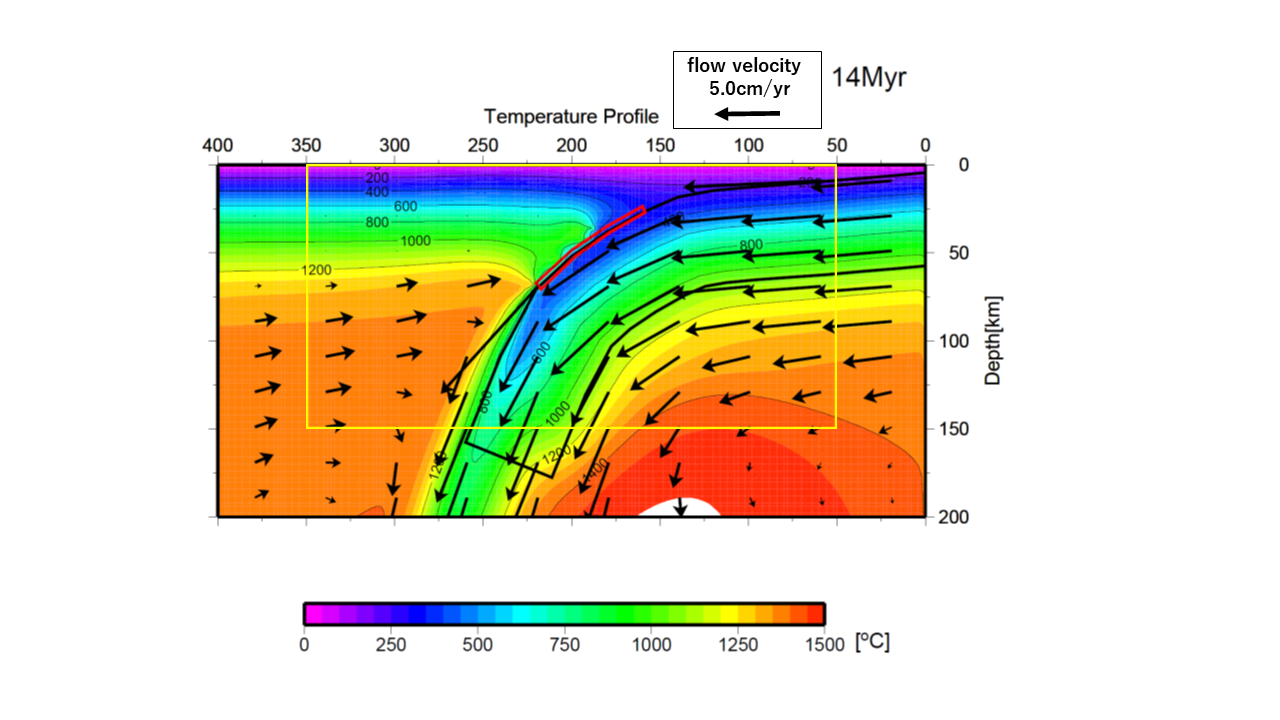
**

(b)

**
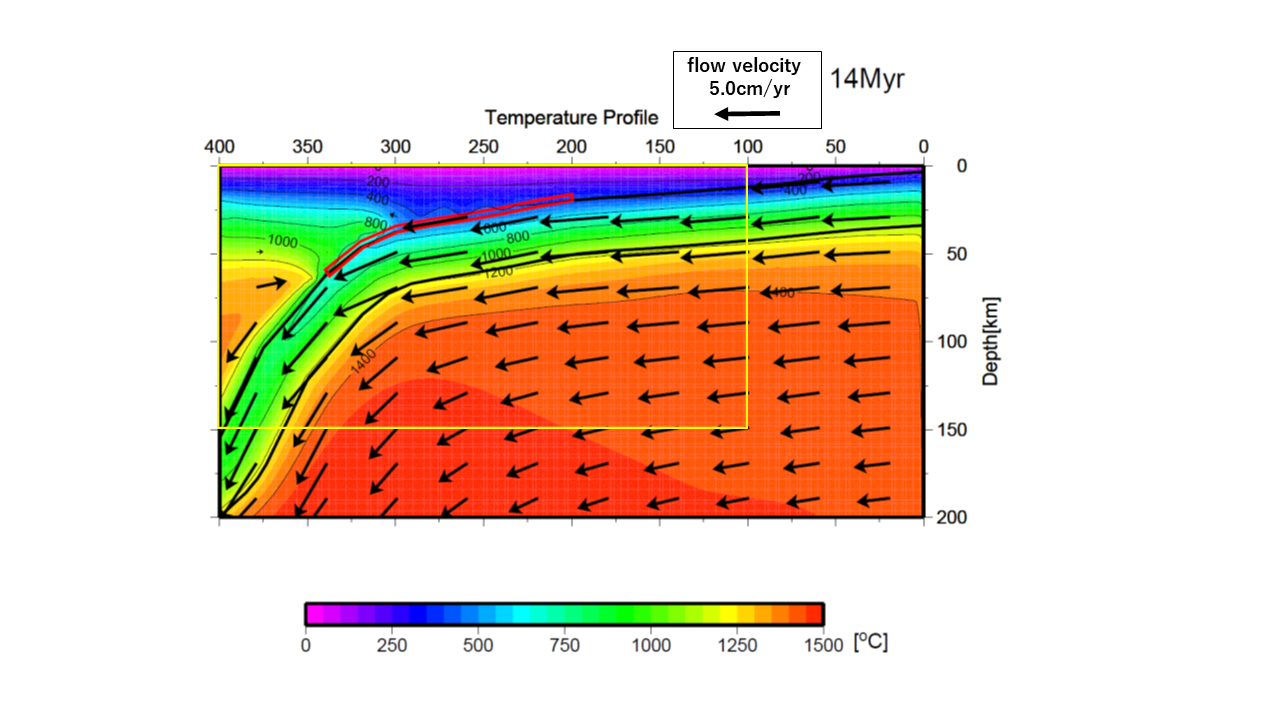
**

**Figure S6** Vertical cross section of the temperature (color) and flow (black arrows) fields associated with subduction of the PHS plate for MODEL II-1 at 0 Ma. The black lines denote the area where the prescribed guide was set, which is bounded by the upper and lower surfaces of the PHS plate. Enclosed area in red denotes the low viscosity layer. Enclosed area in yellow denotes the region where we demonstrate the calculated water content distribution in Fig. 5. (a) Profile A-A’. (b) Profile B-B’.

**Figure S7** Comparison of calculated heat flows for the most suitable model (MODEL II-1) with those for sensitivity test cases along the profile A-A’, which is tabulated in Table S5. Red circles, blue triangles, and pink squares denote the heat flow observed by BSRs*, land borehole and marine heat probe**, and Hi-net observation wells***, respectively. Black solid line denotes calculated heat flows for the best fit model (Fig. S5(b)). Red, green, blue, and yellow dashed lines denote calculated heat flows for CASEs 1, 2, 3-1, and 3-2 in Table S5, respectively.

* Ashi et al. (2002)

** Tanaka et al. (2004); Yamano (2004)

*** Matsumoto (2007)

Ashi, J., Tokuyama, H., and Taira, A., 2002. Distribution of the methane hydrate BSRs and its implication for the prism growth in the Nankai Trough. Marine Geology 3110,1-15.

Tanaka, A., Yamano, M., Yano, Y., and Sasada, M. (2004). Geothermal gradient and heat flow data in and around Japan, digital geoscience map DGN P-5.

Yamano, M. (2004). Heat Flow Data in and around Japan, Digital Geoscience Map DGM P-5, Geological Survey of Japan.

Matsumoto, T., 2007. Terrestrial heat ﬂow anomaly at non-volcanic area in Southwest Japan based on the NIED Hi-net. Japan Geoscience Union Meeting T154-001 (in Japanese).

**Figure S8** Same as Figure S7 except for sensitivity test cases. Black solid line denotes calculated heat flows for the most suitable model (MODEL II-1) (Fig. S5(b)). Red, green, and blue dashed lines denote calculated heat flows for CASEs 4-1, 4-2, and 5 in Table S5, respectively.

**
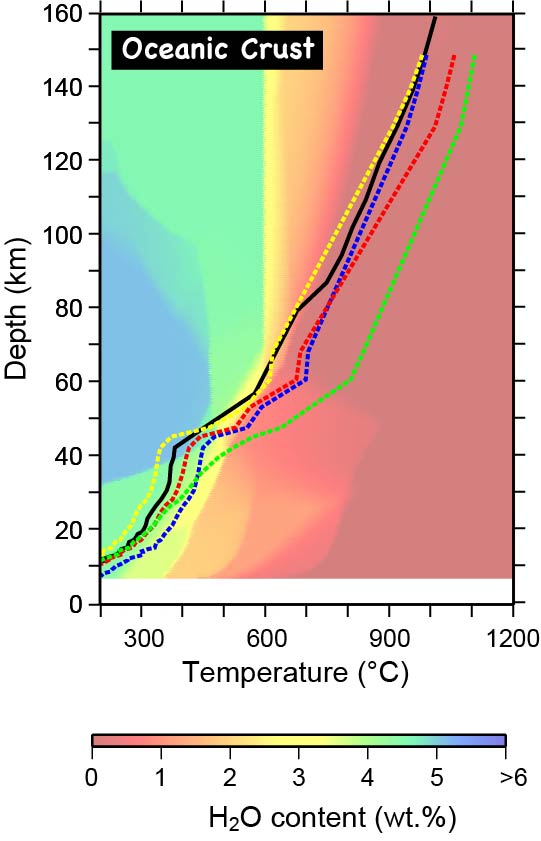
**

**Figure S9** p-T paths along the upper surface of the subducting PHS plate plotted on the phase diagram of water content of MORB calculated from Perple_X. Black solid line denotes p-T path for the most suitable model (MODEL II-1) to the observed heat flow data along the profile A-A’ (Fig. S5(b)). Red, green, blue, and yellow dotted lines denote p-T paths for CASEs 1, 2, 3-1, and 3-2 in Table S5, respectively.

**
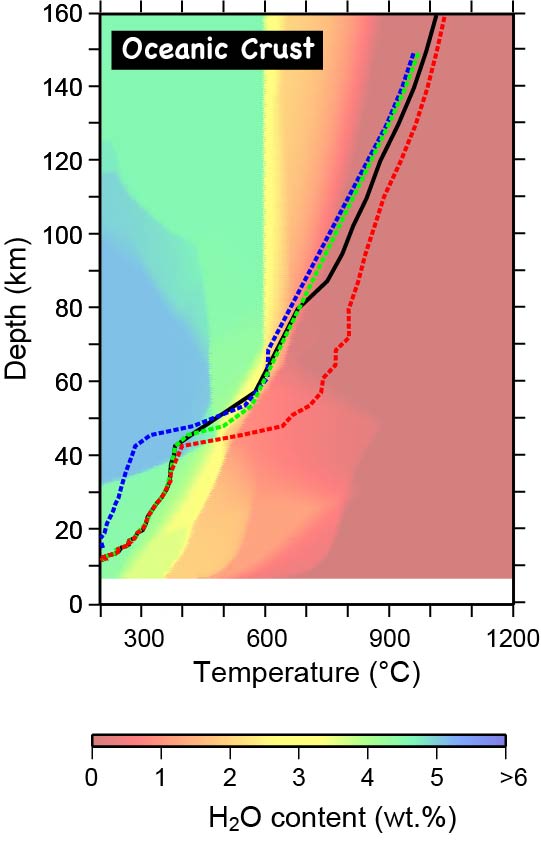
**

**Figure S10** Same as Figure S9 except for sensitivity test CASEs. Black solid line denotes p-T path for the most suitable model (MODEL II-1) to the observed heat flow data along the profile A-A’ (Fig. S5(b)). Red, green, and blue dotted lines denote calculated heat flows for CASEs 4-1, 4-2, and 5 in Table S5, respectively.
